# Considering change

**DOI:** 10.1080/17571472.2017.1410312

**Published:** 2017-12-06

**Authors:** Kate Mtandabari

**Affiliations:** GP, London

**Why this matters to me**

I am passionate about enabling people to articulate what they want to change and feel Coaching tools, in particular, can help people navigate change. Ultimately this is with the aim of helping people fulfil their potential, improve their performance and be happier both in the workplace and their personal lives.

Kate Mtandabari is running a full day Career Development Workshop on Wednesday 7 February 2018 at the Royal College of General Practitioners 30 Euston Square, NW1 2FB (free to RCGP London Members). Book through

www.rcgp.org.uk/learning/london-and-south-england/south-london-faculty/career-development-workshop.aspx.

## Key Messages

There are simple tools available to help those considering change make progress and reach the goals that fit their agenda.

Don’t berate yourself when change not achieved convert it into a learning experience.

Coaching can help clarify the process, increase effectivity of the process and expedite change.

## Personal view

It was a beautiful summer’s afternoon. I remember this because, earlier, I had walked across the river from the South Bank to Charing Cross. It is one of my favourite views – looking down towards St Paul’s on the right, the Houses of Parliament on the left, walking away from the thrill of the SouthBank and joining the mass of activity on the walk from Embankment to Trafalgar Square.

I was in a meeting. The wonderful director I was working with was introducing me to a colleague. ‘When I met Kate it was as a GP’ my boss was saying, ‘but now, of course, I realise she is a change agent’.

This made me stop. I felt this over-riding relief. All these ideas I have, people I connect with and the things I do in my professional and personal life, they are all bound in change.

Change for the better. Change to make a difference. At times, I think it is an aspiration to make life the best it can be, physically or emotionally, for as many people as possible. At others times I recognise something about navigating the turbulent river of the human condition. I could explore why I am interested in change more. And, being psychologically minded, I naturally do. However, most of the time I simply embrace my interest in change and do the ‘doing’ of change. After that meeting, with that wonderful director, on that beautiful summer’s day, that’s exactly what I did. I am still not sure about the words ‘change agent’, but I certainly love what I do and ‘change’ is the theme. 

If you would like to read more about Coaching for change, I have included a brief bibliography and my thoughts about the books below.

John Whitmore’s ‘Coaching for Performance’ (2009) is a gentle introduction to coaching and his GROW model. I found it’s simplicity refreshing and enjoy the book for some light, yet effective, reading.

Many will be familiar with Jenny Roger’s ‘Adult Learning’ text. Her ‘Coaching Skills. A Handbook’ is a deeper consideration of coaching and helped me find Coaching’s meaning and place when I was coming from a therapeutic – clinical background. The NHS is investing in Coaching – to understand what internal caching schemes are about, the strengths and challenges – Katherine St John-Brook’s book is excellent. Finally, so much is being said about resilience and it is such an interesting concept. To consider resilience within different paradigms Carol Pemberton’s book is a great start.

If you would like to explore change further, the approach I have written below uses a coaching paradigm and consists of simple questions. For many, they are good starting point.

## Seven easy steps to change

(1) Think about what you would like to change. What would the ideal ‘changed’ you or your situation look like? Develop some goals.
(2) Consider what’s actually happening for you at the moment. Where is the mismatch between your desired state and now?
(3) Think about how you can move from your ‘now’ to your ‘goal’ state. What options do you have? What resources do you have available? What is within your control?
(4) Break down the changes into manageable and realistic steps. Write them down. Draw them. Share them with someone. Approach them in a way that works best for *you*.
(5) Put some time limits on the steps.
(6) If the changes don’t happen, don’t berate yourself. Show yourself the compassion that you would give another. Stop and consider; what happened? Where were the barriers? Where are the opportunities? Was this actually the change you are looking for?
(7) Consider working with a coach to unpick and action the changes you really want to make. Work with a coach to drive the process through in a time efficient and constructive manner.

## Reflections for those who seek change, but I just don’t feel able to do this

Sometimes people know that they wish to make a change. However, they are simply not emotionally or practically ready or able to take change forward.

This might be due to depression, anxiety or burnout. If you think this might apply to you, show yourself the same empathy you would show a patient. Feeling better is possible and achievable, but it can feel so challenging to take the first steps. Be kind to yourself and seek help either with your GP or one of the agencies below. Tell a loved one. Reach out to a friend.

## Agencies that can help if you are struggling

1. BMA Counselling Service (24hrs)

www.bma.org.uk/advice/work-life-support/your-wellbeing

2. NHS Practitioner Health Programme

www.php.nhs.uk

3. Samaritans

https://www.samaritans.org

4. Your Appraisal Team

## Where to find Coaching

http://www.londonleadershipacademy.nhs.uk

http://www.lpmde.ac.uk/professional-development/coaching-service

Private Providers such as www.katemtandabari.com

RCGP Courses (see below)
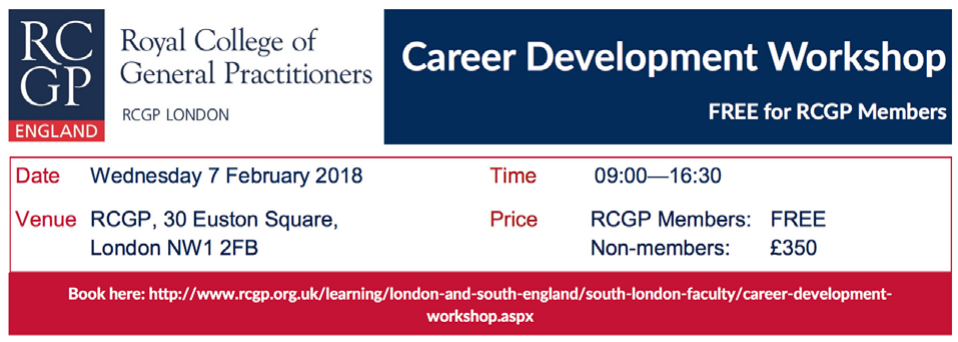


## Disclosure statement

I provide coaching and training for NHS leaders through the NHS Leadership Academy and privately to the corporate sector (www.katemtandabari.com).
